# Acute intraprocedural cardiac tamponade during microwave ablation of hepatocellular carcinoma in segment 2: A case report of emergency percutaneous salvage

**DOI:** 10.1016/j.radcr.2026.04.070

**Published:** 2026-05-28

**Authors:** Stavros K. Grigoriadis, Stavros Spiliopoulos, Konstantinos Palialexis, Dimitrios Filippiadis

**Affiliations:** a Second Department of Radiology, National and Kapodistrian University of Athens, Athens, Greece; b Interventional Oncology Clinic, Athens Medical Centre, Athens, Greece; c Second Department of Radiology, National and Kapodistrian University of Athens, ``Attikon'' University General Hospital, Athens, Greece

**Keywords:** Hepatocellular carcinoma, Microwave ablation, Cardiac tamponade, Hemopericardium, Segment 2 liver, Percutaneous pericardiocentesis

## Abstract

Microwave ablation (MWA) is an established treatment for hepatocellular carcinoma (HCC), but its application in the left hepatic lobe (segment 2) carries a rare but life-threatening risk of thermal injury to the pericardium. We report a case of an 84-year-old male who developed acute cardiac tamponade during MWA of a 2.5 cm HCC. Despite maintaining a safe parenchymal margin and real-time ultrasound monitoring of the needle tip, the patient suffered sudden hemodynamic collapse. Immediate diagnosis via bedside ultrasound and emergency US-guided pericardiocentesis with an 8F catheter placement led to instant stabilization. Follow-up at one month confirmed complete resolution and successful tumor ablation.

Key learning points

• The ``Safety Margin'' Fallacy: A visible parenchymal bridge and real-time needle-tip monitoring may not prevent acute cardiac injury during MWA due to rapid centrifugal expansion of the thermal zone.
• Acute vs. Late Presentation: Unlike delayed inflammatory pericarditis, intraprocedural thermal injury can cause immediate hemodynamic collapse and tamponade requiring bedside intervention.
• Diagnostic Vigilance: Sudden hypotension, tachycardia, and facial cyanosis during subdiaphragmatic ablation should be treated as cardiac tamponade until proven otherwise via immediate bedside ultrasound.
• Interventional Readiness: Interventional Radiologists must have immediate access to pericardiocentesis kits when performing ablations in segment 2 to prevent fatal outcomes.
• Role of Hydrodissection: In high-risk areas like segment 2, hydrodissection should be considered even when a parenchymal margin seems adequate, to serve as an active thermal barrier.

Alt-text: Unlabelled box dummy alt text

## Introduction

Image-guided thermal ablation is a cornerstone in the management of focal liver cancer [1]. While both Radiofrequency Ablation (RFA) and Microwave Ablation (MWA) achieve similar end-point temperatures for coagulative necrosis, MWA is characterized by a more rapid and extensive **active heating volume**[2]. Unlike RFA, MWA is less susceptible to the `heat sink effect' from adjacent vascular structures, which may lead to more aggressive thermal expansion towards the diaphragm and pericardium in subdiaphragmatic locations [[3], [4]]. Consequently, the anatomical proximity of segment 2 to these structures poses unique challenges. While most literature focuses on late-onset pericarditis following thermal injury [5], acute intraprocedural cardiac tamponade is an exceedingly rare and catastrophic event [[6], [7]]. This case underscores the necessity for immediate interventional readiness when treating lesions in high-risk subdiaphragmatic locations.

## Case description

**Fig. 1:**
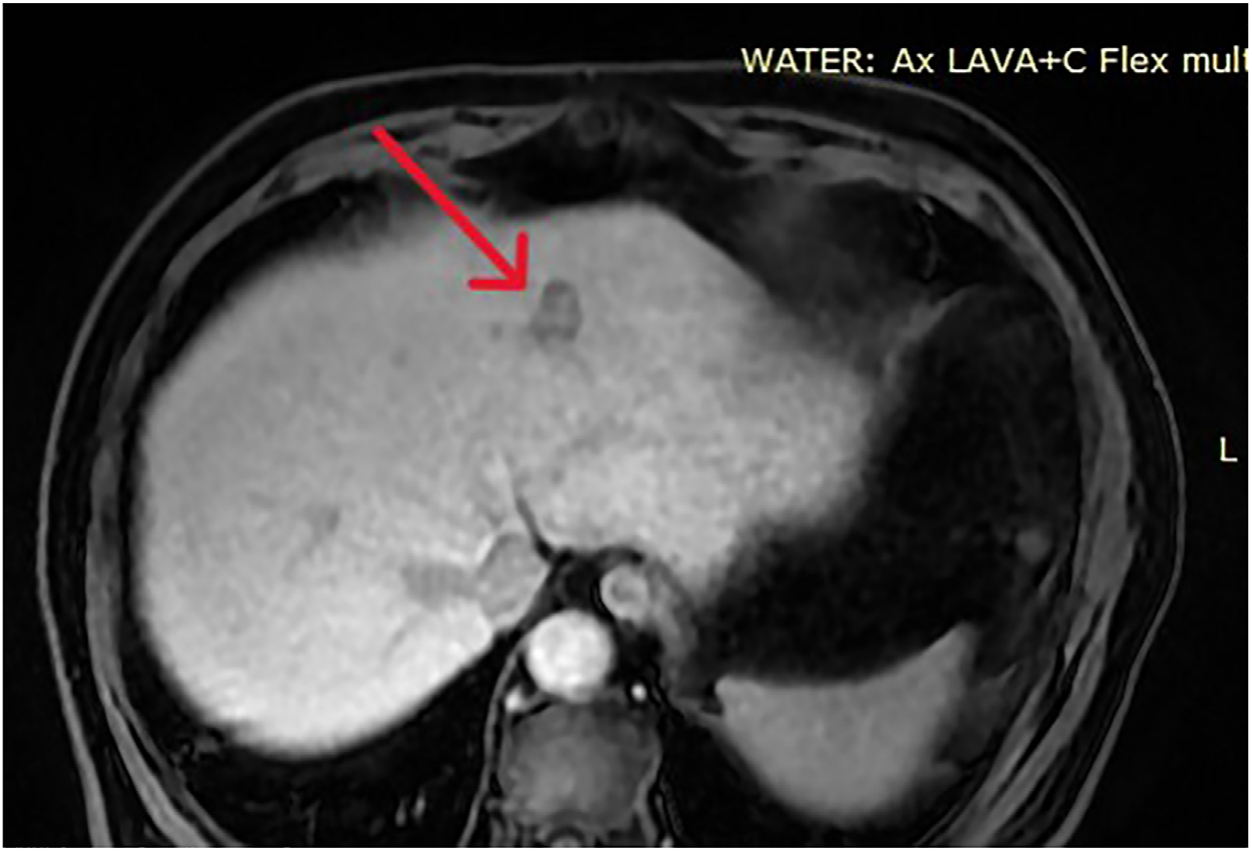
Pre-procedural MRI showing a 2.5 cm HCC in segment 2 (red arrow) with a close anatomical relationship to the pericardium.
• The patient underwent MWA under general anesthesia using combined CT and real-time ultrasound (US) guidance. MWA was selected over Radiofrequency Ablation (RFA) due to its faster heating cycles and reduced susceptibility to the 'heat sink effect' from adjacent large hepatic veins, ensuring a more predictable ablation zone in the cirrhotic liver. During planning, an 8 mm bridge of healthy hepatic parenchyma was identified between the tumor margin and the diaphragm/pericardium. Consequently, hydrodissection was not performed as the distance was deemed safe according to standard protocols.
• The MWA antenna was positioned under real-time US and CT guidance (Fig. 2 (A), Fig. 2 (B)). Toward the end of the ablation cycle, the patient experienced sudden hemodynamic instability: profound hypotension, tachycardia, and facial cyanosis. While ultrasound initially identified a pericardial effusion, an immediate emergency CT was performed to exclude other life-threatening complications, such as active parenchymal bleeding or pneumothorax, and to provide precise anatomical mapping for the subsequent drainage (Fig. 3A).

• An 84-year-old male with NASH-related cirrhosis was diagnosed with a 2.5 cm focal lesion in segment 2 of the liver. MRI demonstrated typical LI-RADS 5 characteristics, including arterial phase hyperenhancement and portal venous phase washout (Fig. 1). Alpha-fetoprotein (AFP) levels were within normal limits. A biopsy confirmed HCC of low-to-moderate differentiation.

**Fig. 2 (A):**
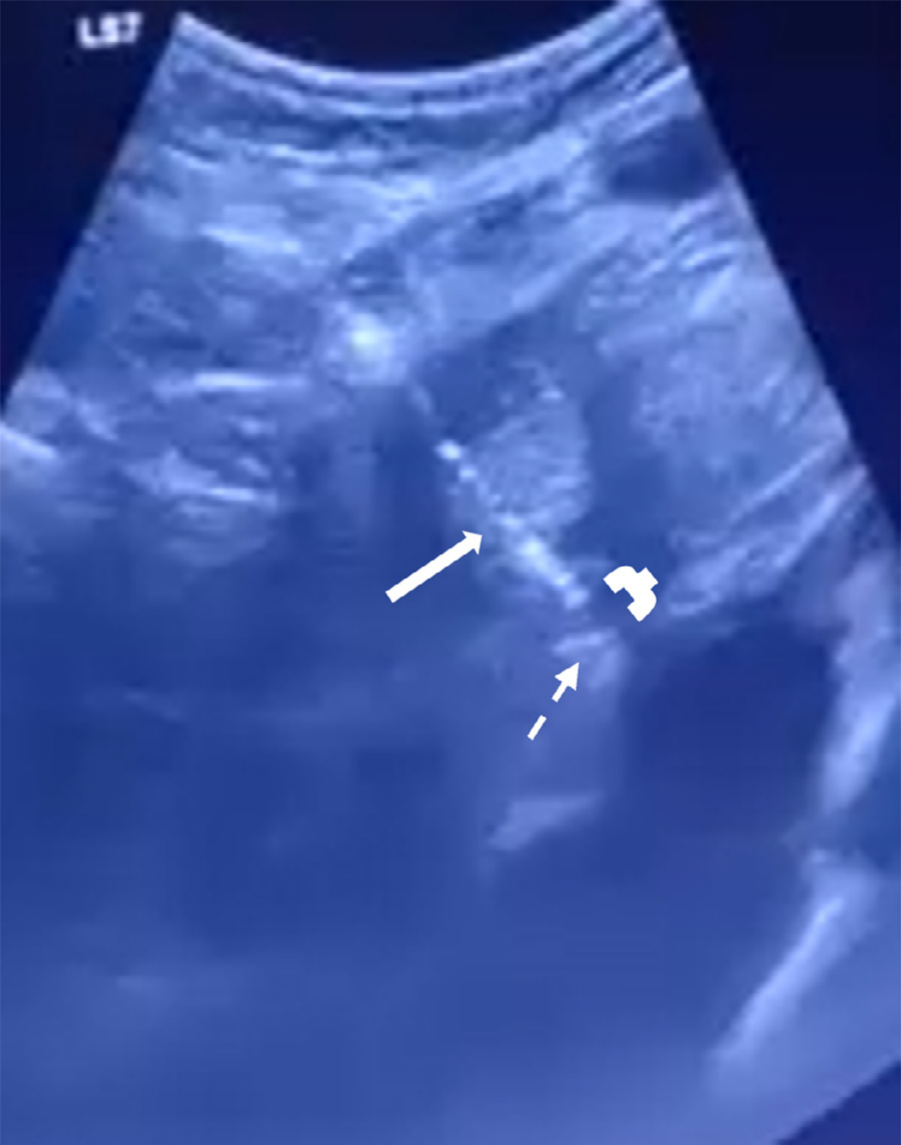
Intraprocedural real-time ultrasound (US) imaging. The white arrow indicates the MWA antenna tip within the lesion. The white bracket denotes the parenchymal bridge. The dotted arrow marks the diaphragmatic contour, demonstrating that that the tip of the antenna (arrowhead) is not penetrating the diaphragm.

**Fig. 2 (B):**
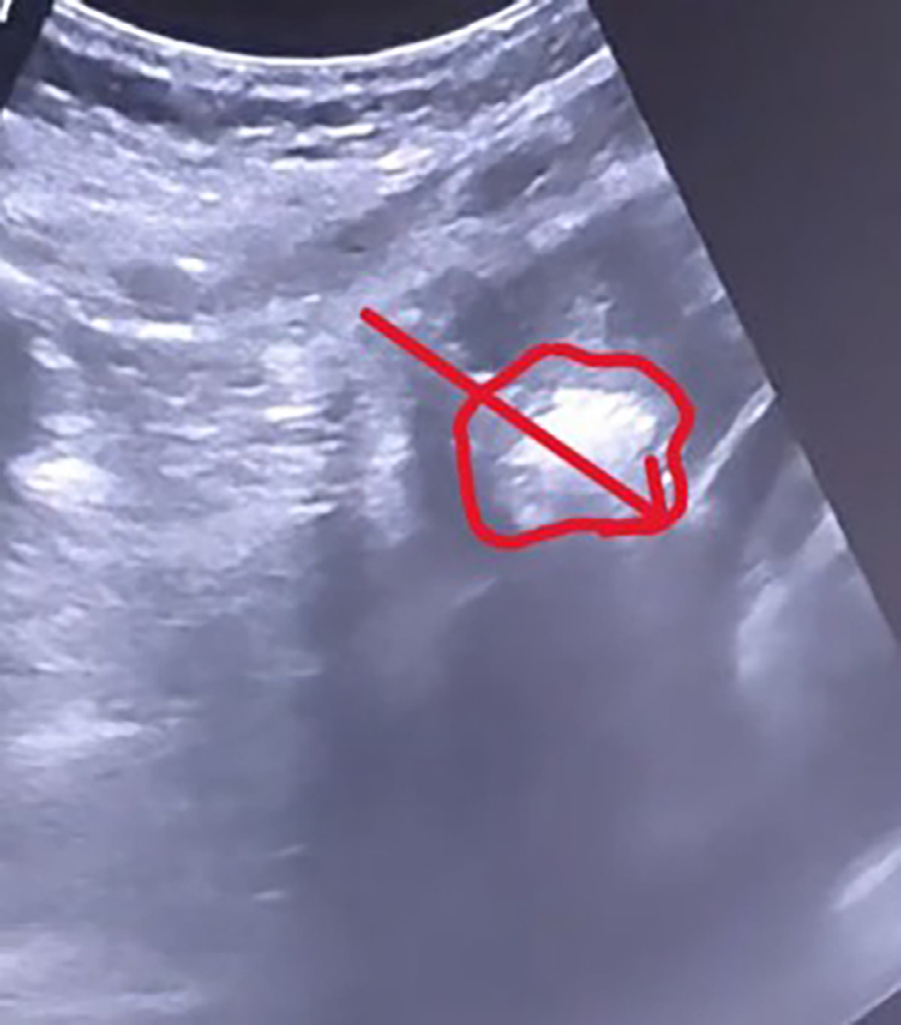
Ablation of the lesion depicting the characteristic hyperechoic imaging. Note that the antenna (arrow) remains in the predetermined position in contact with the diaphragm (denoted in red).

**Fig. 3 (A):**
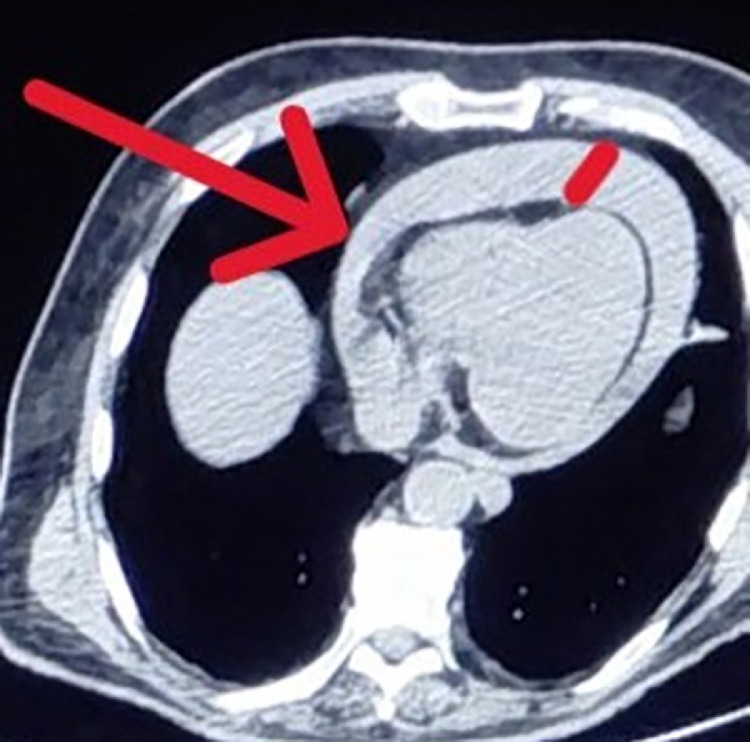
Emergency axial CT performed immediately after hemodynamic collapse. The red arrow points to the large hemopericardium and right heart diastolic collapse (cardiac tamponade).
• Emergency US-guided pericardiocentesis was performed at the bedside. Approximately 160 mL of hemorrhagic fluid was manually aspirated, leading to immediate normalization of vital signs. An 8F pigtail catheter was placed for continuous drainage (Fig. 3B). Post-procedural hemoglobin levels showed a minor drop (from 11.8 g/dL to 10.4 g/dL), which remained stable without transfusion. The patient was discharged after 5 days. A one-month follow-up MRI showed complete technical success with a non-enhancing ablation zone and total resolution of the hemopericardium (Fig. 4).

**Fig. 3 (B):**
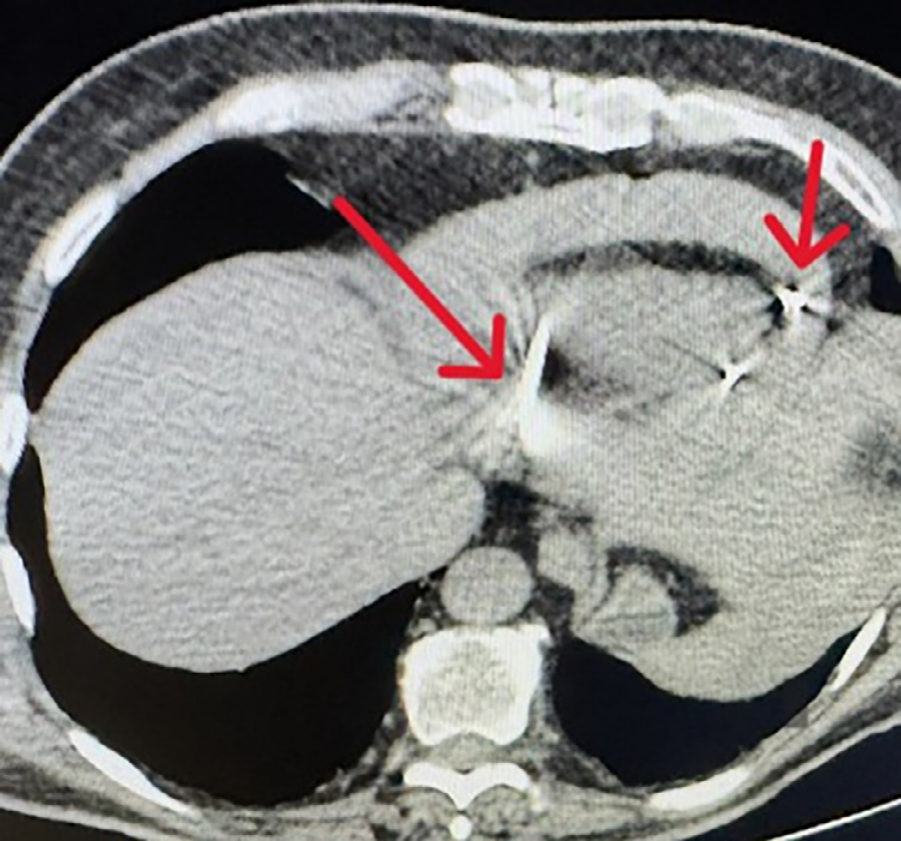
Immediate successful pericardiocentesis with the placement of an 8F pigtail drainage catheter (red arrow).

**Fig. 4:**
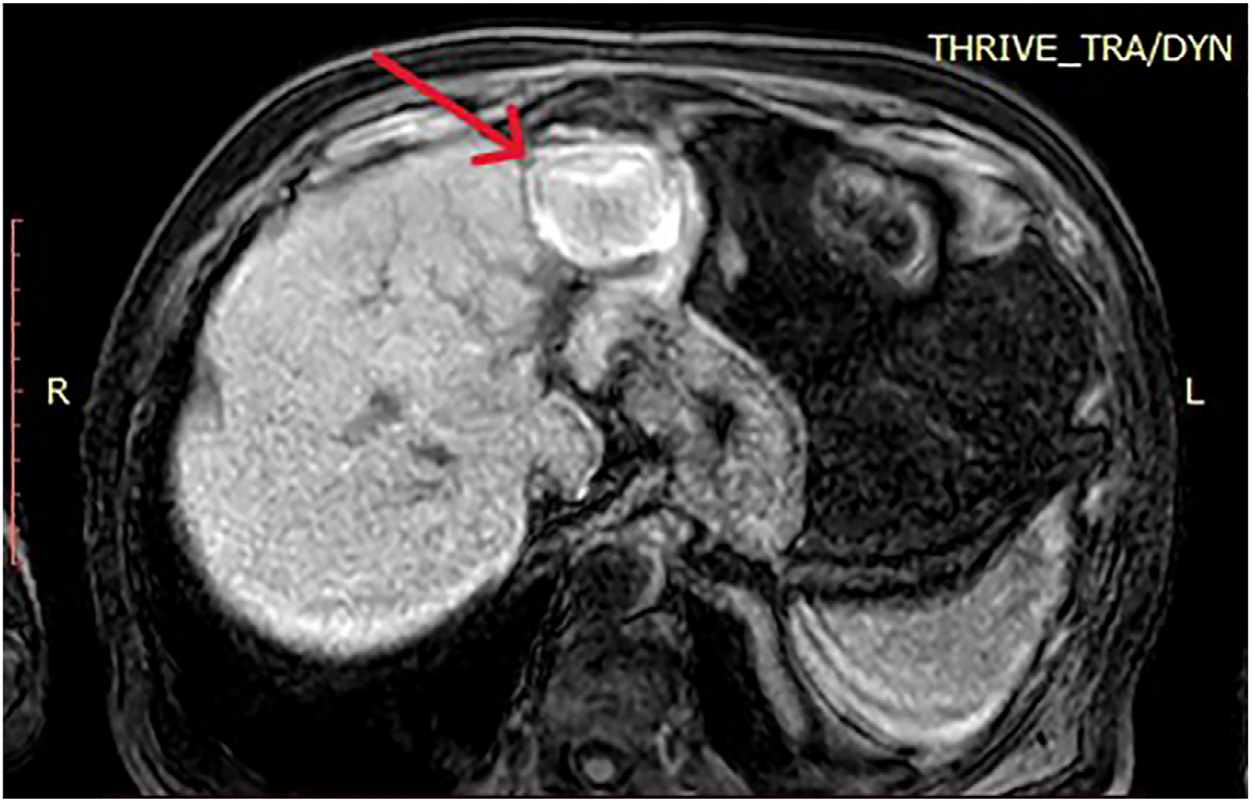
One-month follow-up MRI (Post-contrast T1-weighted) showing complete technical success with a non-enhancing ablation zone (red arrow) and total resolution of hemopericardium.

## Discussion

• The proximity of hepatic segment 2 to the pericardium makes MWA in this location a high-risk procedure. While MWA offers technical advantages, its rapid centrifugal expansion can breach perceived safety margins. In our case, the 8 mm parenchymal bridge proved insufficient. The stability of the antenna under real-time US suggests a trans-diaphragmatic thermal injury rather than mechanical perforation, leading to focal pericardial necrosis and acute tamponade.
• The role of hydrodissection is fundamental. According to SIR and CIRSE clinical guidelines, it should be considered mandatory in segment 2, serving as an active thermal and physical barrier [8]. For tumors where the safety window is extremely narrow, alternative therapies like Y90 Radioembolization (Radiation Segmentectomy) should be evaluated, as they carry a lower risk of acute thermal damage.
• A notable limitation of this case report is the suboptimal quality of certain intraprocedural images. The sudden onset of cardiac tamponade created a life-threatening emergency, requiring the entire interventional team to focus exclusively on the percutaneous salvage of the patient. In such high-stress environments, clinical stabilization takes precedence over imaging documentation or PACS archiving. However, this ``real-world'' documentation serves as a critical teaching point for Interventional Radiologists regarding the urgency and gravity of this rare complication.

## Conclusion

Interventional radiologists should maintain a high index of suspicion for cardiac tamponade when patients exhibit sudden hemodynamic collapse during subdiaphragmatic ablations. Although a parenchymal bridge exists, the aggressive nature of MWA thermal zones may necessitate the routine use of hydrodissection as an additional thermal barrier in segment 2. This case also underscores the necessity for interventional radiologists to have immediate access to pericardiocentesis kits during subdiaphragmatic ablations. Prompt recognition and emergency percutaneous salvage are the only means to prevent a fatal outcome when the ``safety margin'' fails.

## Patient consent

Informed consent for publication of this case report and any accompanying images was obtained from the patient. A copy of the written consent is available for review by the Editor-in-Chief of this journal.
